# THE EFFECT OF PATIENT-DRIVEN PAYMENT MODEL REIMBURSEMENT POLICY CHANGE ON NURSING HOMES

**DOI:** 10.1093/geroni/igad104.1546

**Published:** 2023-12-21

**Authors:** Gregory Orewa, Robert Weech-Maldonado, Ganisher Davlyatov, Sue Feldman, David Becker, Justin Lord

**Affiliations:** University of Texas, San Antonio, San Antonio, Texas, United States; University of Alabama at Birmingham, Birmingham, Alabama, United States; University of Oklahoma Health Sciences Center, Oklahoma City, Oklahoma, United States; University of Alabama at Birmingham, Birmingham, Alabama, United States; University of Alabama at Birmingham, Birmingham, Alabama, United States; Louisiana State University at Shreveport, Shreveport, Louisiana, United States

## Abstract

To promote more value in skilled nursing facility spending in the US, CMS modified the way it pays for nursing homes (NH) services on October 1, 2019. The new Medicare reimbursement methodology, the Patient Driven Payment Model (PDPM), moves away from the traditional system focused on therapist minutes to a value-based focused on resident clinical characteristics. Using contingency theory, this study explores NH responses to PDPM by: 1) examining whether NH’s therapist and nursing staffing patterns are aligning with the policy incentives; and 2) whether the adjustments are resulting in improved financial performance. Seven different datasets were merged for 2017– 2021: Medicare cost reports, Payroll Based Journal, NH Compare, Area Health Resource File, HHS Provider Relief Fund, CDC NH COVID-19 public file, and CDC COVID-19 Data Tracker. The data was modelled using random-effects regression, and we tested for potential moderation effect of staffing on the relationship between PDPM and financial performance. Dependent variables were nursing and therapy staffing intensity, while the independent variable was PDPM for model 1. Dependent variable was operating margin, moderator variable was staffing intensity, and independent variable was PDPM for model 2. Organizational, community level, and COVID-19 related variables were used as controls. Results suggest that PDPM was associated with an increase in RN and LPN staffing intensity, but a decrease in Physical Therapist, Physical Therapist Assistant, and Occupational Therapist Assistant staffing intensity (p< 0.05). PDPM had a positive impact on financial performance moderated by RN and PT staffing intensity (p< 0.05). Policy and managerial implications are discussed.

